# Antimicrobial resistance in fecal *Escherichia coli* and *Salmonella enterica* isolates: a two-year prospective study of small poultry flocks in Ontario, Canada

**DOI:** 10.1186/s12917-019-2187-z

**Published:** 2019-12-21

**Authors:** Csaba Varga, Michele T. Guerin, Marina L. Brash, Durda Slavic, Patrick Boerlin, Leonardo Susta

**Affiliations:** 1 Department of Pathobiology, College of Veterinary Medicine, University of Illinois at Urbana-Champaign, Urbana, Illinois 61802 USA; 2 Department of Population Medicine, Ontario Veterinary College, University of Guelph, Guelph, Ontario N1G 2W1 Canada; 3 Animal Health Laboratory, University of Guelph, Guelph, Ontario N1G 2W1 Canada; 4 Department of Pathobiology, Ontario Veterinary College, University of Guelph, Guelph, Ontario N1G 2W1 Canada

**Keywords:** Antimicrobial susceptibility, Multidrug resistance, Chicken, Turkey, Poultry, Backyard flock, Antibiotic, Surveillance, Longitudinal study

## Abstract

**Background:**

Although keeping small poultry flocks is increasingly popular in Ontario, information on the antimicrobial susceptibility of enteric bacteria of such flocks is lacking. The current study was conducted on small poultry flocks in Ontario between October 2015 and September 2017, and samples were submitted on a voluntary basis to Ontario’s Animal Health Laboratory. From each submission, a pooled cecal sample was obtained from all the birds of the same species from the same flock and tested for the presence of two common enteric pathogens, *E. coli* and *Salmonella.* Three different isolates from each *E. coli*-positive sample and one isolate from each *Salmonella*-positive sample were selected and tested for susceptibility to 14 antimicrobials using a broth microdilution technique.

**Results:**

A total of 433 fecal *E. coli* isolates (358 chicken, 27 turkey, 24 duck, and 24 game bird) and 5 *Salmonella* isolates (3 chicken, 1 turkey, and 1 duck) were recovered. One hundred and sixty-seven chicken, 5 turkey, 14 duck, and 15 game bird *E. coli* isolates were pan-susceptible. For *E. coli*, a moderate to high proportion of isolates were resistant to tetracycline (43% chicken, 81% turkey, 42% duck, and 38% game bird isolates), streptomycin (29% chicken, 37% turkey, and 33% game bird isolates), sulfonamides (17% chicken, 37% turkey, and 21% duck isolates), and ampicillin (16% chicken and 41% turkey isolates). Multidrug resistance was found in 37% of turkey, 20% of chicken, 13% of duck, and 8% of game bird *E. coli* isolates. *Salmonella* isolates were most frequently resistant to streptomycin, tetracycline, and sulfonamides. Resistance to cephalosporins, carbapenems, macrolides, and quinolones was infrequent in both *E. coli* and *Salmonella* isolates. Cluster and correlation analyses identified streptomycin-tetracycline-sulfisoxazole-trimethoprim-sulfamethoxazole as the most common resistance pattern in chicken *E. coli* isolates. Turkey *E. coli* isolates compared to all the other poultry species had higher odds of resistance to tetracycline and ampicillin, and a higher multidrug resistance rate.

**Conclusions:**

*Escherichia coli* isolates were frequently resistant to antimicrobials commonly used to treat poultry bacterial infections, which highlights the necessity of judicious antimicrobial use to limit the emergence of multidrug resistant bacteria.

## Background

Non-commercial poultry flocks (denoted as “small flocks”) are increasingly popular in urban, suburban, and rural areas in North America [1–3]. Small flocks can pose a health risk to their owners by exposing them to zoonotic pathogens [4–7] through consumption of contaminated meat or eggs [8, 9], or direct contact with infected birds [10] or their environment [11]. Antimicrobial resistance (AMR) in zoonotic pathogens adds to this risk [12–15] because infections with antimicrobial resistant bacteria are more difficult to treat, and result in higher morbidity and mortality [16, 17]. Inappropriate antimicrobial use has been shown to be one of the main causes for the development of AMR in commensal and pathogenic bacteria of poultry [17, 18]. Exposure to an individual antimicrobial may cause the bacteria to develop resistance to multiple antimicrobials if resistance genes are located on mobile genetic elements [19]. Furthermore, these acquired resistance determinants can persist even after the antibiotic selection pressure ends [12].

Health Canada categorizes antimicrobials based on their importance in human medicine: I - very high importance; II - high importance; III - medium importance; and IV - low importance [20]. The classification system considers the antimicrobial’s indication (e.g., preferred choice for treatment of serious human infections) and availability of replacements (e.g., limited substitutes available) [20]. Under an amended Canadian regulation that came into effect on December 1, 2018, all medically important antimicrobials (Categories I, II, and III) used in food animals require a veterinary prescription in order to help limit the development and spread of AMR [21].

In Canada, AMR of *Escherichia coli* and *Salmonella* isolates obtained from commercial broiler chicken and turkey flocks is monitored by the Canadian Integrated Program for Antimicrobial Resistance Surveillance [22]. Emergence of resistance to antimicrobials commonly used to treat bacterial infections in commercial poultry flocks in Ontario is well documented [22–24]. In contrast, only one study [25], which was conducted in provincially inspected slaughter plants, has documented resistance to antimicrobials in small flocks in Ontario. Thus, the objectives of this study were to evaluate AMR patterns of fecal *E. coli* and *Salmonella enterica* isolates of chickens, turkeys, waterfowl, and game birds from Ontario small flocks submitted for laboratory diagnostic testing because of morbidity or mortality, and to determine differences in AMR patterns among different poultry species.

## Results

### Description of submissions

Over the 2-year period, the Animal Health Laboratory received 160 small flock submissions, with a median of 1 bird per submission (range = 1–5), from flocks ranging in size from 1 to 299 birds (median 25) and birds ranging in age from 6 days to 7 years (median 7 months). The majority of submissions were chickens (84%, 134 submissions), although a few turkey (10), duck (8), and game bird (8) submissions were also received [26].

### Antimicrobial resistance of *Salmonella* isolates

Of 159 submissions tested for *Salmonella* spp. (a sample from one chicken submission was not available), a total of 5 isolates were recovered (5 pooled samples, 1 isolate recovered from each pooled sample). Serotypes included *S.* Anatum, *S. Indiana*, and *S.* Ouakam (3 chicken pooled samples), *S.* Uganda (1 turkey pooled sample), and *S.* Montevideo (1 duck pooled sample) [26]. Three *Salmonella* isolates were pan-susceptible (1 *S. Indiana*, 1 *S.* Montevideo, and 1 *S.* Uganda). The *S.* Anatum isolate was resistant to streptomycin, and the *S.* Ouakam isolate was multidrug resistant (streptomycin-sulfisoxazole-trimethoprim-sulfamethoxazole-tetracycline).

### Antimicrobial resistance of *E. coli* isolates

Of 159 submissions tested for fecal *E. coli*, a total of 433 isolates were recovered: 358 from chicken submissions (120 pooled samples; 3 isolates recovered from 119 pooled samples and 1 isolate recovered from 1 pooled sample); 27 from turkey submissions (9 pooled samples, 3 isolates recovered from each pooled sample); 24 from duck submissions (8 pooled samples, 3 isolates recovered from each pooled sample); and 24 from game bird submissions (8 pooled samples, 3 isolates recovered from each pooled sample). Of these, 46.65% of the chicken (167/358), 18.52% of the turkey (5/27), 58.33% of the duck (14/24), and 62.50% of the game bird (15/24) isolates were pan-susceptible.

In the chicken *E. coli* isolates, there was a high frequency of resistance (≥40% of isolates) to tetracycline, a moderate frequency of resistance (15–39% of isolates) to streptomycin, sulfisoxazole, and ampicillin, and a low frequency of resistance (5–14% of isolates) to trimethoprim-sulfamethoxazole and gentamicin (Table 1). All of the other antimicrobials tested had a very low frequency of resistance (< 5%). In the turkey *E. coli* isolates, there was a high frequency of resistance to tetracycline and ampicillin, and a moderate frequency of resistance to streptomycin and sulfisoxazole. In the duck *E. coli* isolates, there was a high frequency of resistance to tetracycline, and a moderate frequency of resistance to sulfisoxazole and trimethoprim-sulfamethoxazole. In the game bird *E. coli* isolates, there was a moderate frequency of resistance to tetracycline and streptomycin.

**Table 1:** Percentage of fecal *Escherichia coli* isolates from Ontario small poultry flocks that were resistant to 14 selected antimicrobials, as determined by a broth microdilution technique, by poultry species

Antimicrobial class	Antimicrobial^A^	Chicken (*N* = 358)	Turkey (*N* = 27)	Duck (*N* = 24)	Game birds (*N* = 24)
		n (%)^B^ [CI]^C^	n (%) [CI]	n (%) [CI]	n (%) [CI]
Aminoglycosides	GEN	23 (6.42) [4.12–9.48]	1 (3.70) [0.09–18.97]	0	0
	STR	105 (29.33) [24.66–34.34]	10 (37.04) [19.40–57.63]	2 (8.33) [1.03–27.00]	8 (33.33) [15.63–55.32]
β-Lactams	AMP	57 (15.92) [12.29–20.13]	11 (40.74) [22.39–61.20]	1 (4.17) [0.11–21.12]	1 (4.17) [0.11–21.12]
	AMC	8 (2.23) [0.97–4.36]	2 (7.41) [0.91–24.29]	0	0
	CRO	4 (1.12) [0.31–2.84]	2 (7.41) [0.91–24.29]	0	0
	FOX	5 (1.40) [0.46–3.23]	1 (3.70) [0.09–18.97]	0	0
	MER	0	0	0	0
Folate inhibitors	SSS	61 (17.04) [13.29–21.34]	10 (37.04) [19.40–57.63]	5 (20.83) [7.13–42.15]	2 (8.33) [1.03–27.00]
	STX	30 (8.38) [5.73–11.75]	3 (11.11) [2.35–29.16]	4 (16.67) [4.74–37.38]	0
Macrolides	AZM	5 (1.40) [0.46–3.23]	0	2 (8.33) [1.03–27.00]	0
Phenicols	CHL	17 (4.75) [2.79–7.49]	3 (11.11) [2.35–29.16]	1 (4.17) [0.11–21.12]	0
Quinolones	CIP	1 (0.28) [0.007–1.55]	3 (11.11) [2.35–29.16]	1 (4.17) [0.11–21.12]	0
	NAL	8 (2.23) [0.97–4.36]	3 (11.11) [2.35–29.16]	1 (4.17) [0.11–21.12]	0
Tetracyclines	TET	155 (43.30) [38.10–48.61]	22 (81.48) [61.92–93.70]	10 (41.67) [22.11–63.36]	9 (37.50) [18.80–59.41]

^A^*GEN* gentamicin, *STR* streptomycin, *AMP* ampicillin, *AMC* amoxicillin-clavulanic acid, *CRO* ceftriaxone, *FOX* cefoxitin, *MER* meropenem, *SSS* sulfisoxazole, *STX* trimethoprim-sulfamethoxazole, *AZM* azithromycin, *CHL* chloramphenicol, *CIP* ciprofloxacin, *NAL* nalidixic acid, *TET* tetracycline

^B^Number and percentage of isolates resistant to the antimicrobial

^C^CI = Exact binomial 95% confidence interval for the percentage of isolates resistant to the antimicrobial

In the chicken *E. coli* isolates, the most common AMR patterns were ampicillin-streptomycin-tetracycline (22 isolates, 6.15%) and streptomycin-tetracycline (19 isolates, 5.31%) (Table 2). The latter was also common in the game bird *E. coli* isolates.

**Table 2:** Most common antimicrobial resistance patterns of fecal *Escherichia coli* isolates from Ontario small poultry flocks, by poultry species

Poultry species	Antimicrobial resistance pattern^A^	Number of antimicrobial classes in pattern (multidrug resistant)^B^	n (%)^C^
Chicken (*N* = 358)	STR-TET	2 (no)	19 (5.31)
	AMP-STR-TET	3 (yes)	22 (6.15)
	SSS-STR-TET	3 (yes)	6 (1.68)
	GEN-SSS-STR	2 (no)	6 (1.68)
	GEN-SSS-STR-TET	3 (yes)	5 (1.40)
	AMP-SSS-STR-STX-TET	4 (yes)	5 (1.40)
Turkey (*N* = 27)	AMP-TET	2 (no)	3 (11.11)
	STR-TET	2 (no)	2 (7.41)
	SSS-STR-TET	3 (yes)	2 (7.41)
	AMP-SSS-STR-STX-TET	4 (yes)	2 (7.41)
	AMP-CHL-CIP-NAL-SSS-STR-TET	6 (yes)	2 (7.41)
Duck (*N* = 24)	SSS-STX-TET	2 (no)	2 (8.33)
Game bird (*N* = 24)	STR-TET	2 (no)	6 (25.00)
	SSS-STR-TET	3 (yes)	2 (8.33)

^A^Resistance to 14 selected antimicrobials (including amoxicillin-clavulanic acid, ceftriaxone, cefoxitin, meropenem, azithromycin), as determined by a broth microdilution technique. *GEN* gentamicin, *STR* streptomycin, *AMP* ampicillin, *SSS* sulfisoxazole, *STX* trimethoprim-sulfamethoxazole, *CHL* chloramphenicol, *CIP* ciprofloxacin, *NAL* nalidixic acid, *TET* tetracycline

^B^An isolate was defined as multidrug resistant if it was non-susceptible to at least one antimicrobial in ≥3 antimicrobial classes (Aminoglycosides: GEN, STR; β-Lactams: AMP; Folate biosysnthesis pathway inhibitors: SSS, STX; Phenicols: CHL; Quinolones: CIP, NAL; Tetracyclines: TET)

^C^Number and percentage of isolates with each antimicrobial resistance pattern. For chicken, only patterns with ≥5 isolates are shown, and for other poultry species, only patterns with ≥2 isolates are shown

Multidrug resistance was detected in 19.55% (95% CI = 15.57–24.05) of the chicken, 37.04% (95% CI = 19.40–57.63) of the turkey, 12.50% (95% CI = 2.66–32.36) of the duck, and 8.33% (95% CI = 1.03–27.00) of the game bird *E. coli* isolates.

**Fig. 1:**
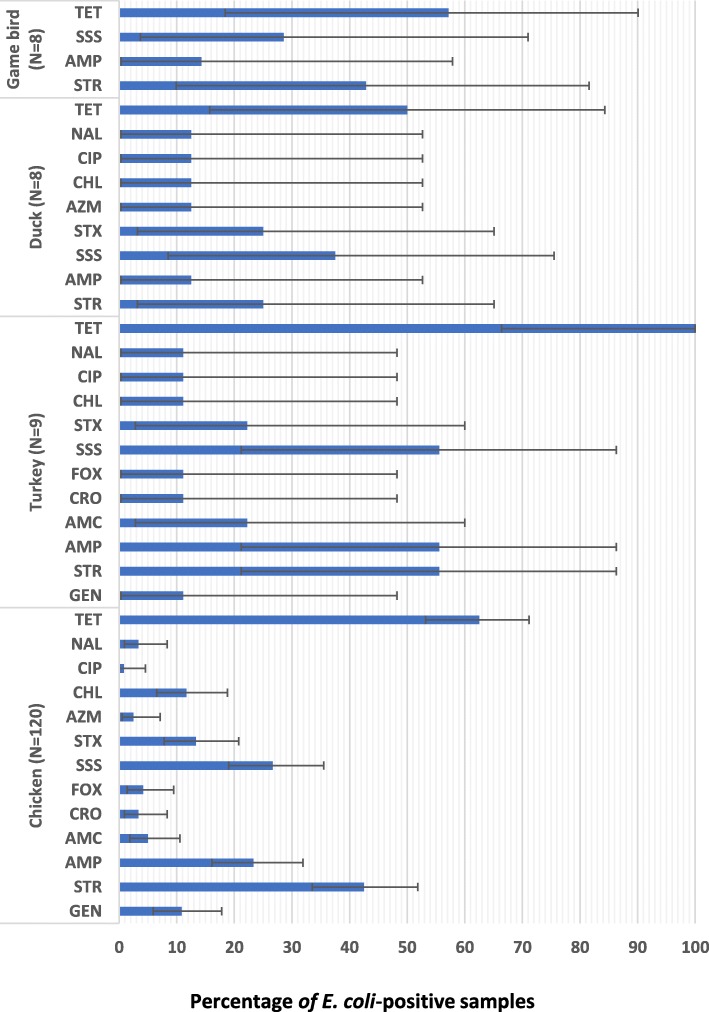
Percentage of *Escherichia coli*-positive fecal samples from Ontario small poultry flocks with production, clinical illness, or mortality issues that were resistant to antimicrobials, by poultry species.^ABCD^. ^A^Sample-level resistance to 14 selected antimicrobials (including meropenem), as determined by a broth microdilution technique. GEN = gentamicin; STR = streptomycin; AMP = ampicillin; AMC = amoxicillin-clavulanic acid; CRO = ceftriaxone; FOX = cefoxitin; SSS = sulfisoxazole; STX = trimethoprim-sulfamethoxazole; AZM = azithromycin; CHL = chloramphenicol; CIP = ciprofloxacin; NAL = nalidixic acid; TET = tetracycline. Only antimicrobials for which resistance was detected are shown. ^B^Antimicrobial classes. Aminoglycosides (GEN, STR); β-Lactams (AMP, AMC, CRO, FOX); Folate biosynthesis pathway inhibitors (SSS, STX); Macrolides (AZM); Phenicols (CHL); Quinolones (CIP, NAL); Tetracyclines (TET). ^C^For each poultry species, estimates were computed by dividing the number of samples resistant to an antimicrobial by the total number of *E. coli*-positive samples. A sample was considered to be resistant to a specific antimicrobial if at least one isolate from a pooled cecal sample was resistant. ^D^Exact binomial 95% confidence interval for the proportion of antimicrobial resistant samples

A high (≥40%) proportion of *E. coli*-positive samples were resistant to tetracycline (62.50% of the chicken, 100% of the turkey, 50% of the duck, and 57.14% of the game bird samples), streptomycin (42.50% of the chicken, 55.56% of the turkey, and 42.86% of the game bird samples), ampicillin (55.56% of the turkey samples), and sulfisoxazole (55.56% of the turkey samples) (Fig. 1).

**Fig. 2:**
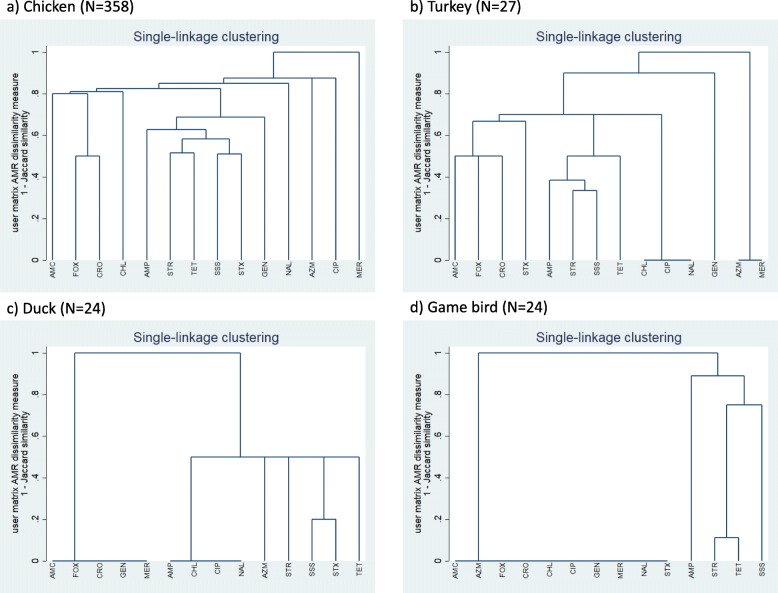
Single-linkage clustering dendrograms of resistance of fecal *Escherichia coli* isolates to antimicrobials, by poultry species^ABC^. ^A^GEN = gentamicin; STR = streptomycin; AMP = ampicillin; AMC = amoxicillin-clavulanic acid; CRO = ceftriaxone; FOX = cefoxitin; MER = meropenem; SSS = sulfisoxazole; STX = trimethoprim-sulfamethoxazole; AZM = azithromycin; CHL = chloramphenicol; CIP = ciprofloxacin; NAL = nalidixic acid; TET = tetracycline. ^B^A cluster analysis, using the Jaccard binary similarity coefficient, was used to compare individual antimicrobials with respect to their similarity in the resistance status of *E. coli*. The Jaccard distance measures dissimilarity between antimicrobials and is obtained by subtracting the Jaccard binary similarity coefficient from one [27]. A high dissimilarity measure indicates that relatively few isolates were resistant to both antimicrobials, a low dissimilarity measure indicates that a relatively high proportion of isolates were resistant to both antimicrobials, and a dissimilarity measure of zero indicates that all isolates were susceptible to both antimicrobials

Single-linkage clustering dendrograms with Jaccard distances for *E. coli* resistance are presented in Fig. 2. A relatively high proportion (i.e., a cluster) of the chicken *E. coli* isolates were resistant to streptomycin, tetracycline, sulfisoxazole, and trimethoprim-sulfamethoxazole; a second cluster of chicken *E. coli* isolates was resistant to cefoxitin and ceftriaxone. Other notable clusters included resistance to streptomycin, sulfisoxazole, ampicillin, and tetracycline (turkey *E. coli* isolates), amoxicillin-clavulanic acid, cefoxitin, and ceftriaxone (turkey *E. coli* isolates), sulfisoxazole and trimethoprim-sulfamethoxazole (duck *E. coli* isolates), and streptomycin and tetracycline (game bird *E. coli* isolates). The turkey, duck, and game bird *E. coli* isolates were pan-susceptible to several antimicrobials.

The strongest, statistically significant pairwise correlations with respect to resistance of *E. coli* at the isolate-level (chicken isolates only) were between cefoxitin and ceftriaxone (ρ = 0.67), sulfisoxazole and trimethoprim-sulfamethoxazole (ρ = 0.67), streptomycin and sulfisoxazole (ρ = 0.51), streptomycin and tetracycline (ρ = 0.49), gentamicin and sulfisoxazole (ρ = 0.49), and ampicillin and streptomycin (ρ = 0.46) (Table 3). All pairwise relationships were positive.

**Table 3:** Pairwise correlations between antimicrobials (with respect to resistance) of fecal *Escherichia coli* isolates of chickens from Ontario small poultry flocks (*n* = 358)^AB^

	AMC	AMP	AZM	FOX	CRO	CHL	CIP	GEN	NAL	SSS	STR	STX	TET
AMC	1.000												
AMP	0.347	1.000											
AZM	–	–	1.000										
FOX	0.304	–	0.189	1.000									
CRO	0.344	0.244	0.214	0.667	1.000								
CHL	0.322	0.262	0.197	0.197		1.000							
CIP	–	–	–	–	–	0.237	1.000						
GEN	0.192	–	–	–	0.189	–	–	1.000					
NAL	–	–	–	–	–	–	0.350		1.000				
SSS	0.283	0.310	–	–	–	0.283	–	0.487	0.183	1.000			
STR	0.193	0.458	–	–	–	–	–	0.332	0.235	0.508	1.000		
STX	0.295	0.364	0.223	–	–	0.264	–	–	0.295	0.667	0.315	1.000	
TET	–	0.329	–	–	–	–	–	–	–	0.294	0.490	0.204	1.000

^A^*GEN* gentamicin, *STR* streptomycin, *AMP* ampicillin, *AMC* amoxicillin-clavulanic acid, *CRO* ceftriaxone, *FOX* cefoxitin, *MER* meropenem, *SSS* sulfisoxazole, *STX* trimethoprim-sulfamethoxazole, *AZM* azithromycin, *CHL* chloramphenicol, *CIP* ciprofloxacin, *NAL* nalidixic acid, *TET* tetracycline

^B^Spearman rank correlation, with a Bonferroni correction (α/14) to adjust for multiple comparisons, was used to measure the strength and direction of the relationships between individual antimicrobials with respect to resistance of *E. coli* at the isolate-level (chicken isolates only). Only statistically significant (*P* ≤ 0.0036) correlations are shown

### Differences in AMR of fecal *E. coli* isolates between poultry species

The odds of resistance to tetracycline (odds ratio = 5.89, 95% CI = 1.71–20.29, *P* = 0.005) and ampicillin (odds ratio = 4.06, 95% CI = 1.24–13.25, *P* = 0.020) were significantly higher in turkey *E. coli* isolates compared to isolates from all the other poultry species. The rate of MDR was significantly higher (incidence rate ratio = 1.99, 95% CI = 1.16–3.40, *P* = 0.012) in turkey *E. coli* isolates compared to isolates from all the other poultry species.

## Discussion

Our study evaluated AMR in fecal *E. coli* and *Salmonella enterica* isolates from birds from small flocks experiencing morbidity, mortality, or production issues, and found a relatively high frequency of resistance to antimicrobials commonly used to treat bacterial infections in poultry. Differences in flock characteristics, including poultry species, health status (diseased or healthy), or husbandry (commercial or non-commercial), or dissimilarities in study design, analytical methods (isolate-level or flock-level analysis), sampling protocols (on-farm, at slaughter plants, or at diagnostic laboratories), or antimicrobial susceptibility testing (disk diffusion or broth microdilution) can make comparisons to other studies difficult. Our comparisons are limited to results from studies that evaluated AMR in fecal *E. coli* and *Salmonella* spp. in small flocks using samples collected on-farm, at diagnostic laboratories, or at slaughter.

Outbreaks of human salmonellosis linked to contact with small flocks have been reported in the United States [4, 28], Bangladesh [29], and Chile [30]. The *Salmonella* serotypes identified in our study (Anatum, Indiana, Ouakam, Uganda, and Montevideo) are not among the most prevalent commercial poultry-associated serotypes (Kentucky, Enteritidis, Heidelberg, and Typhimurium) in Canada [31] or the United States [32], and they are less frequently associated with human salmonellosis cases in Canada [33]. Nonetheless, the presence of AMR in *Salmonella* in small flocks is concerning because of the risk that resistant salmonellae pose in human cases with bacteremia or a compromised immune system. Although the frequency of *Salmonella enterica* was very low in our study [26], and many of the *Salmonella* isolates were pan-susceptible, some were resistant to streptomycin alone, or to streptomycin, sulfisoxazole, trimethoprim-sulfamethoxazole, and tetracycline. Our findings agree with a recent study that evaluated AMR in *Salmonella* isolated from small flock cases at the California Animal Health and Food Safety Laboratory System, and found resistance to streptomycin, sulfonamides, and tetracycline [34].

In the fecal *E. coli* isolates in our study, there was a very low frequency of resistance to cephalosporins, carbapenems, macrolides, and quinolones, which are antimicrobials classified in Canada as being of very high or high importance in human medicine [20]. This is an encouraging finding from a human health viewpoint because flock owners can be exposed to antimicrobial resistant zoonotic pathogens [6, 13, 15] through direct contact with their birds [10] or their environment [11], or consumption of contaminated meat or eggs [8, 9]. However, there was a moderate to high frequency of resistance to tetracycline, streptomycin, sulfonamides, and ampicillin; antimicrobials frequently used to treat bacterial infections in poultry [35]. These findings were in accordance with other small flock studies in Canada (tetracycline resistance 37%, streptomycin 21%, sulfisoxazole 16%, ampicillin 15%) [25] and Ecuador (tetracycline 69%, streptomycin 42%, sulfisoxazole 65%, ampicillin 45%) [36], and one study of commercial broiler chicken flocks in Canada (tetracycline 53%, streptomycin 33%, sulfisoxazole 18%, ampicillin 38%) [23].

Our cluster and correlation analyses of the chicken *E. coli* isolates showed that there was concurrent resistance to streptomycin, tetracycline, sulfisoxazole, and trimethoprim-sulfamethoxazole; clusters of turkey, duck, and game bird isolates included many of the same antimicrobials. Our cluster analyses also showed that there was concurrent resistance to cefoxitin and ceftriaxone in the chicken *E. coli* isolates and to amoxicillin-clavulanic acid, cefoxitin, and ceftriaxone in the turkey *E. coli* isolates. The moderate frequency of MDR in the turkey and chicken *E. coli* isolates (and to a lesser extent in the duck and game bird *E. coli* isolates), and the strong correlations in resistance of the chicken *E. coli* isolates to antimicrobials commonly used to treat bacterial infections of poultry, highlight the importance of judicious antimicrobial use to limit the development and dissemination of multidrug resistant bacteria in small flocks [12, 13].

Our regression models showed that there were higher probabilities of resistance to tetracycline and ampicillin in the *E. coli* isolates of turkeys when compared to isolates obtained from all the other poultry species. Moreover, the rate of MDR was significantly higher in the turkey isolates compared to all the other species. Differences in AMR between poultry species might be explained by variation in antimicrobial use or husbandry practices. However, these findings should be interpreted cautiously because chicken isolates were over-represented in our study. Therefore, further studies are needed to assess factors that might have a role in the development of AMR in commensal and pathogenic enteric bacteria of small flocks.

Limitations of this study include a sampling bias, as most submissions came from southwestern and eastern Ontario, which might have been the consequence of the geographic proximity to the diagnostic laboratories in Guelph and Kemptville, respectively [26]. Also, small flocks were not randomly selected, and our study included only owners who had a flock veterinarian, as this is a laboratory submission requirement. We also used fecal samples from diagnostic submissions and not from healthy birds. Our study might therefore overestimate the frequency of AMR because samples came from birds that might have already been treated with antimicrobials.

## Conclusions

Our study enhances the knowledge on AMR of small flocks by evaluating the AMR patterns of *E. coli* and *Salmonella* isolates from chickens, turkeys, ducks, and game birds. These results can be used as a benchmark for ongoing monitoring of AMR in enteric bacteria of small flocks in Ontario, in light of the recently amended antimicrobial use regulation in Canada. Ultimately, the findings derived from this study can be used to educate veterinarians and small flock owners about issues surrounding AMR, with a goal of reducing the presence of multidrug resistant bacteria in small flocks and mitigating the risk they might pose to public health.

## Methods

### Study design

Samples were obtained through a prospective surveillance study of small flocks conducted in Ontario between October 2015 and September 2017, which is described in detail elsewhere [26]. In brief, a small flock was defined as a non-commercial poultry flock composed of not more than 299 broiler chickens, 99 layer chickens, 49 turkeys, 300 waterfowl, or 300 game birds. Small flock owners who had issues with production, clinical illness, or mortality in their flock were provided the opportunity to submit birds for diagnostic testing for a discounted fee. Submissions (*n* = 160) were made to the Animal Health Laboratory, University of Guelph through the owner’s veterinarian. A maximum of 5 sick and/or dead birds of one species from the same flock constituted a submission. Live birds submitted to the lab were euthanized using carbon dioxide.

### Sample collection and bacterial isolation

All bacterial isolation and antimicrobial susceptibility testing were conducted at the Animal Health Laboratory, Guelph, Ontario. From each submission, one pooled cecal sample was collected (from all of the birds of the same species from the same flock in the submission) and tested for fecal *E. coli* and *Salmonella* spp. Cecal material was directly plated on MacConkey and Hektoen enteric agars (Oxoid Ltd., Nepean, ON) for *E. coli* isolation, and inoculated into buffered peptone water (Bio-Media Unlimited Ltd., Toronto, ON) for *Salmonella* spp. pre-enrichment. Aliquots of buffered peptone water were then transferred to Hajna tetrathionate (Animal Health Laboratory, Guelph, ON) and Rappaport Vasiliadis broths (Bio-Media Unlimited Ltd.) for *Salmonella* spp. enrichment, followed by plating on brilliant green (Bio-Media Unlimited Ltd.) and XLT-4 agars (Oxoid Ltd.). Presumptive *E. coli* and *Salmonella* spp. colonies were identified using matrix-assisted laser desorption ionization time-of-flight mass spectrometry (Bruker Ltd., Billerica, MA) [37]. *Salmonella*-positive isolates were submitted to the OIE (World Organisation for Animal Health) *Salmonella* reference laboratory at the National Microbiology Laboratory in Guelph for serotyping according to published methods [38].

### Antimicrobial susceptibility testing and classification

Three different isolates from each *E. coli*-positive sample and one isolate from each *Salmonella*-positive sample were purposively selected. Susceptibility testing of *E. coli* and *Salmonella* isolates to 14 antimicrobials was conducted using automated broth microdilution (Sensititre®; Trek Diagnostic Systems Inc., Westlake, OH) with the National Antimicrobial Monitoring System CMV4AGNF panel [22]. Based on the interpretive standards of the Canadian Integrated Program for Antimicrobial Resistance Surveillance [22], *E. coli* and *Salmonella* isolates with a minimum inhibitory concentration lower than or equal to the Susceptible breakpoint were classified as *susceptible*, whereas those with a minimum inhibitory concentration higher than the Susceptible breakpoint were considered to be *resistant*. The Susceptible breakpoints are: amoxicillin-clavulanic acid (≤ 8/4 μg/mL); ampicillin (≤ 8 μg/mL); azithromycin (≤ 16 μg/mL); cefoxitin (≤ 8 μg/mL); ceftriaxone (≤ 1 μg/mL); chloramphenicol (≤ 8 μg/mL); ciprofloxacin (≤ 0.06 μg/mL); gentamicin (≤ 4 μg/mL); meropenem (≤ 1 μg/mL); nalidixic acid (≤ 16 μg/mL); streptomycin (≤ 16 μg/mL); sulfisoxazole (≤ 256 μg/mL); tetracycline (≤ 4 μg/mL); and trimethoprim-sulfamethoxazole (≤ 2/38 μg/mL) [22].

An isolate was defined as multidrug resistant if it was non-susceptible to at least one antimicrobial in ≥3 different antimicrobial classes [39]. In our study, classes included: Aminoglycosides (gentamicin, streptomycin); β-Lactams (amoxicillin-clavulanic acid, ampicillin, cefoxitin, ceftriaxone, meropenem); Folate biosynthesis pathway inhibitors (sulfisoxazole, trimethoprim-sulfamethoxazole); Macrolides (azithromycin); Phenicols (chloramphenicol); Quinolones (ciprofloxacin, nalidixic acid); and Tetracyclines (tetracycline).

### Data analysis

Antimicrobial susceptibility data were entered into a spreadsheet (Microsoft Excel 2016, Microsoft Corporation, Redmond, WA), reviewed for missing values, and subsequently transferred into a statistical software program (STATA Intercooled, version 14.2, Stata Corporation, College Station, TX) for analysis.

For each poultry species (chicken, turkey, duck, and game bird), estimates of the proportion of *E. coli* and *Salmonella* isolates that were resistant to each of the 14 tested antimicrobials were computed by dividing the number of isolates resistant to an antimicrobial by the total number of isolates tested for the antimicrobial. In addition, estimates of the proportion of isolates that showed multidrug resistance (MDR) were computed by dividing the number of multidrug resistant isolates by the total number of isolates tested.

Further, for each poultry species, estimates of the percentage of *E. coli*-positive samples that were resistant to each of the 14 tested antimicrobials were computed by dividing the number of samples resistant to an antimicrobial by the total number of *E. coli*-positive samples. A sample was considered to be resistant to a specific antimicrobial if at least one isolate from a pooled cecal sample was resistant. For all estimates, exact binomial 95% confidence intervals (CIs) were calculated.

To compare individual antimicrobials with respect to their similarity in the resistance status of *E. coli*, a cluster analysis, using the Jaccard binary similarity coefficient, was performed for each poultry species. The number of isolates that are resistant to both antimicrobials and the number that are resistant to one yet susceptible to the other are used in the calculation of the coefficient. Dendrograms were constructed using the single-linkage clustering method with the Jaccard distance. The Jaccard distance measures dissimilarity between antimicrobials and is obtained by subtracting the Jaccard binary similarity coefficient from one [27]. Thus, a high dissimilarity measure indicates that relatively few isolates were resistant to both antimicrobials, whereas a low dissimilarity measure indicates that a relatively high proportion of isolates were resistant to both antimicrobials. A dissimilarity measure of zero indicates that all isolates were susceptible to both antimicrobials.

Further, to measure the strength and direction of the relationships between individual antimicrobials with respect to resistance of *E. coli* at the isolate-level, Spearman’s rank correlation coefficients were calculated; only chicken isolates were evaluated. A Bonferroni correction was used to adjust for multiple comparisons among antimicrobials, with *P* ≤ 0.0036 (α of 0.05/14) indicating a significant correlation.

To identify differences in *E. coli* resistance between poultry species, logistic regression was used; only antimicrobials for which ≥5% of the isolates were resistant were evaluated. Therefore, 6 of 14 antimicrobials were analyzed: ampicillin, gentamicin, streptomycin, sulfisoxazole, trimethoprim-sulfamethoxazole, and tetracycline. Four population-averaged models were built for each antimicrobial using the generalized estimating equation method, with a robust variance estimate and exchangeable correlation structure to account for sample-level clustering. In these univariable models, the binary (yes/no) dependent variable represented the frequency of resistance to the antimicrobial, while the independent variable was poultry species (binary variable: chickens compared to all the other poultry species; turkeys compared to all the other poultry species; ducks compared to all the other poultry species; and game birds compared to all the other poultry species). This method of grouping species together was preferred over analyzing species as a categorical variable because of the limited number of isolates from turkeys, ducks, and game birds. A *P*-value ≤0.05 on the Wald *χ*^2^ test indicated a statistically significant association.

In addition, four Poisson regression models were built to identify differences in *E. coli* MDR between poultry species using the generalized estimating equation method described above. The dependent variable was the number of antimicrobial classes to which an isolate was resistant; as seven antimicrobial classes were studied, this count potentially ranged from zero to seven. The independent variable was the poultry species (binary variable; described above).

## Data Availability

The datasets generated and/or analyzed during the current study are not publicly available, as the data will be used for additional epidemiological analyses.

## Abbreviations

AMR: Antimicrobial resistance
CI: Confidence interval
MDR: Multidrug resistance
